# Investigation of the causal etiology in a patient with T-B+NK+ immunodeficiency

**DOI:** 10.3389/fimmu.2022.928252

**Published:** 2022-07-29

**Authors:** Robert Sertori, Jian-Xin Lin, Esteban Martinez, Sadhna Rana, Andrew Sharo, Majid Kazemian, Uma Sunderam, Mark Andrake, Susan Shinton, Billy Truong, Roland M. Dunbrack, Chengyu Liu, Rajgopol Srinivasan, Steven E. Brenner, Christine M. Seroogy, Jennifer M. Puck, Warren J. Leonard, David L. Wiest

**Affiliations:** ^1^Blood Cell Development and Function Program, Fox Chase Cancer Center, Philadelphia, PA, United States; ^2^Laboratory of Molecular Immunology, Immunology Center, National Heart, Lung, and Blood Institute, National Institutes of Health (NIH), Bethesda, MD, United States; ^3^Innovation Labs, Tata Consultancy Services, Hyderabad, India; ^4^Center for Computational Biology, University of California, Berkeley, CA, United States; ^5^Departments of Biochemistry and Computer Science, Purdue University, West Lafayette, IN, United States; ^6^Molecular Therapeutics Program, Fox Chase Cancer Center, Philadelphia, PA, United States; ^7^Transgenic Core, National Heart, Lung, and Blood Institute, National Institutes of Health (NIH), Bethesda, MD, United States; ^8^Department of Pediatrics, University of Wisconsin School of Medicine and Public Health, Madison, WI, United States; ^9^Department of Pediatrics, University of California San Francisco and UCSF Benioff Children’s Hospital, San Francisco, CA, United States

**Keywords:** immunodeficiency, newborn screening, zebrafish, thymus, MED14, T cell lymphopenia, severe combined immunodeficiency (SCID)

## Abstract

Newborn screening for severe combined immunodeficiency (SCID) has not only accelerated diagnosis and improved treatment for affected infants, but also led to identification of novel genes required for human T cell development. A male proband had SCID newborn screening showing very low T cell receptor excision circles (TRECs), a biomarker for thymic output of nascent T cells. He had persistent profound T lymphopenia, but normal numbers of B and natural killer (NK) cells. Despite an allogeneic hematopoietic stem cell transplant from his brother, he failed to develop normal T cells. Targeted resequencing excluded known SCID genes; however, whole exome sequencing (WES) of the proband and parents revealed a maternally inherited X-linked missense mutation in *MED14 (MED14^V763A^)*, a component of the mediator complex. Morpholino (MO)-mediated loss of MED14 function attenuated T cell development in zebrafish. Moreover, this arrest was rescued by ectopic expression of cDNA encoding the wild type human *MED14* ortholog, but not by *MED14^V763A^*, suggesting that the variant impaired MED14 function. Modeling of the equivalent mutation in mouse (*Med14^V769A^)* did not disrupt T cell development at baseline. However, repopulation of peripheral T cells upon competitive bone marrow transplantation was compromised, consistent with the incomplete T cell reconstitution experienced by the proband upon transplantation with bone marrow from his healthy male sibling, who was found to have the same *MED14^V763A^* variant. Suspecting that the variable phenotypic expression between the siblings was influenced by further mutation(s), we sought to identify genetic variants present only in the affected proband. Indeed, WES revealed a mutation in the L1 cell adhesion molecule *(L1CAM^Q498H^)*; however, introducing that mutation *in vivo* in mice did not disrupt T cell development. Consequently, immunodeficiency in the proband may depend upon additional, unidentified gene variants.

## Introduction

Primary immune deficiencies are rare, with severe combined immunodeficiency (SCID) occurring approximately 1/66,000 live births in the United States (1). SCID is defined as the absence of T lymphocytes and absent or nonfunctional B lymphocytes (2). Historically, SCID was diagnosed when patients manifested life-threatening infections in the first few months of life (3); however, in 2005, a newborn screening approach was developed that enabled reliable identification of patients with SCID prior to the onset of infections (4). The newborn screening assay measures the presence of T cell receptor excision circles (TRECs) in dried blood spots from peripheral blood. TRECs are a biproduct of T cell receptor (TCR) gene rearrangement and constitute a biomarker for normal T cell development in the thymus. TREC-based newborn screening, now adopted throughout the United States and several countries, afforded two significant benefits. First, earlier diagnosis of SCID enables the initiation of treatment prior to the onset of infection, thereby markedly increasing treatment efficacy (5, 6). Second, TREC screening has facilitated efforts to establish the molecular etiology of T cell lymphopenic conditions, leading to identification of a number of novel regulators of T cell development (7–10). Specifically, identifying the causative mutation in a T lymphopenic patient entails the targeted resequencing of known immunodeficiency genes to determine if disease results from a mutation in a known gene. Upon exclusion of known causes, whole exome sequencing (WES) is performed on the patient and parents to identify candidate variants, which then must be functionally studied to identify the causal variant. The zebrafish model is useful to evaluate human candidate variants, having high conservation of genes and processes controlling hematopoiesis and immune cell development (11) and ease of genetic manipulation through direct injection of embryos (12).

Here we describe a male proband identified by newborn screening as having low TRECs and reduced T lymphocytes. After exclusion of known causes of immunodeficiency, WES revealed a missense mutation in a component of the Mediator Complex, *MED14*, which is inherited in an X-linked manner. The multiprotein mediator complex is required for gene transcription by RNA polymerase II, and has been shown to influence epigenetic regulation, transcriptional elongation, termination, mRNA processing, noncoding RNA activation, and super-enhancer formation, making it a critical regulator of development and lineage determination (13, 14). MED14 functions as a backbone of the complex, and loss of MED14 is lethal (15, 16). Functional screening of the patient MED14 variant suggested that it may have contributed to the patient’s disease.

## Materials and methods

### Human subjects and genomic analysis

Immunodeficiency was identified in the male proband by routine newborn SCID screening of a blood filter card (17). Research activities were performed with parental informed consent under protocols approved by the institutional review boards (IRBs) at the University of California, San Francisco and National Heart, Lung, and Blood Institute (NHLBI), National Institutes of Health (NIH), Bethesda, MD. Research-based WES was performed on cells from the patient and parents with bioinformatic analysis and variant calling as described (7). Additional WES was performed using genomic DNA (gDNA) from EBV lines derived from the patient and his parents and PBMC from his healthy brother. Briefly, gDNA (3 μg) was fragmented by sonication to generate 100-500 bp fragments. The DNA fragments were then end-repaired, 3’ dA overhangs were added, and adaptors were ligated per the manufacturer’s instructions. After removing free adaptors using Agencourt AMPure XP beads, the DNA fragments were amplified by 6 cycles of PCR. The exons and UTRs were enriched using Exon V4 plus UTR (SureSelect^XT^ Target Enrichment System for Illumina, Agilent Technologies); the enriched DNA was additionally amplified by 12 cycles of PCR, and 250-400 bp fragments were purified by 2% E-gel (ThermoFisher) and Gel Purification Kit (Zymo Research) and sequenced on a HiSeq platform (Illumina). Sequencing reads were mapped to hg19 using Bowtie2 and BWA using default parameters. Aligned reads were piled up by Samtools Mpileup using the following parameters: (-A -B -Q 30 -q 20 -d 10000 -L 1000 -h 50 -o 10 -e 17 -m 3). The output was converted to VCF file using Bcftools (view -vcg) and was converted to Annovar format for annotation using Convert2annovar.pl. All common variants (AF>0.0001) in the ExAC database were filtered. Remaining variants were annotated using Annovar, and non-exonic variants were removed. Nonsynonymous variants were categorized as X-linked, de-novo, or autosomal recessive (AR). X-linked variants hemizygous in the proband and heterozygous or absent in the mother were examined, as were autosomal de-novo variants absent in either parent and AR variants that were heterozygous in each parent, heterozygous or absent in the unaffected sibling, but homozygous or compound heterozygous in the proband.

### Animals

Tuebingen long fin zebrafish were maintained at 28.5°C under standard aquaculture conditions. Animal housing and handling were all performed in accordance with the approved protocols from the Fox Chase Cancer Center Institutional Animal Care and Use Committee (IACUC). Likewise, mouse experiments were performed under the auspices of IACUC-approved animal protocols, and all mouse strains were housed in accredited facilities at either Fox Chase Cancer Center or NIH. All experiments using mice at NHLBI were performed using protocols approved by the NHLBI Animal Care and Use Committee and followed NIH guidelines for use of animals in intramural research.

### Ortholog analysis

Genomic sequences were obtained by searching the NCBI and ENSEMBL databases. Multiple alignments of human, mouse and zebrafish MED14 and SMARCAL1 amino acid sequences were obtained using Clustal X. Clustal X was also used for a multiple species alignment of human, mouse, rat, bovine, frog, zebrafish, worm and fly sequences.

### Structural modeling

To assess the extent to which the V763A MED14 patient variant alters MED14 structure, we performed structural modeling by surveying all PDB structures that contain MED14, including the following PDB codes: 7EMF, 7ENA, 7ENC, 7ENJ (18), 7LBM (19), and 7NVR (representative of 9 other companion structures from the same paper) (20). The PDB structure code 7ENA (MED14 is author chain n) was chosen as the representative structure. The topology and overall conformation of amino acids 637-884 of the protein were conserved for all the structures examined, making it suitable for a computational analysis of the missense change V763A. In the MED14 fragment containing amino acids 637-884, the Valine at position 763 was substituted with Alanine using a backbone dependent rotamer library employed in the UCSF Chimera 1.15 software package (21, 22). Both the wildtype and V763A versions of this MED14 fragment were subjected to two different protein relaxation methods using the Rosetta molecular modeling suite to optimize side chain packing and to obtain an energy score that would reflect the stability of the V763A variant compared to wildtype MED14 (23). Either a coordinate-constrained method of relaxation or a full atom relax were employed alone or in combination, to allow for backbone movement in addition to side chain packing steps (24, 25). The stability of the V763A variant compared to wildtype was assessed by the All-Atom Rosetta energy scoring function of the conserved fragment from amino acids 637 to 884 (26). The same modeling analysis was also performed on the murine equivalent (V769A) to the human V763A MED14 variant. As there were two chain breaks in the coordinates of the mouse MED14 in the PDB entry 6W1S (chain I), we submitted the equivalent fragment of mouse MED14 (residues 643 to 890) to the ColabFold advanced version python notebook (27).

### Zebrafish experiments

The zebrafish orthologs of candidate patient variants, MED14 (*med14*; NM_212765.2) and SMARCAL1 (*smarcal1*; NM_001127466.1), were identified by homology and synteny as described (28). We designed and obtained antisense morpholino (MO) oligonucleotides to block the pre-mRNA splicing of zebrafish *med14* and *smarcal1* from Gene Tools (**Table 1**). MO dose was established by injecting titrated quantities of MO into one-cell zebrafish embryos, following which MO efficacy was assessed by reverse-transcriptase (RT)–PCR as described using the indicated primers (**Table 1**) (12, 29). The effect of MO knockdown on T cell development was assessed by whole mount *in situ* hybridization (WISH) as described (30), using the following probes: *lck, ikaros, tcrd* and *foxn1* (28, 31). The stained embryos were photographed using a Nikon SMZ1500 stereomicroscope equipped with DS-Fi1 digital camera and Nikon Ar imaging software. Image J software was used to measure integrated staining density of zebrafish thymi. Experiments to assess the capacity of wild type and patient variant MED14 (V763A) to rescue the arrest of T cell development caused by MO depletion of endogenous *med14* comprised heat-inducible re-expression as described (7). Wild type and patient variant (V763A) human *MED14* constructs were produced using Vector Builder, sequenced, and subcloned into pSGH2. Ectopic expression of wild-type and mutant human MED14 was achieved by injection of the heat-inducible pSGH2 vector into one-cell–stage embryos (32), following which re-expression was induced by elevating the temperature to 37°C for 1 hour at 30 hours post fertilization (hpf). GFP^+^ embryos in which re-expression of *MED14* was induced were selected at 5 days post fertilization (dpf) for analysis by WISH using an lck probe. Image J software was used to measure integrated staining density of zebrafish thymi.

**Table 1:** Oligonucleotides used in this study.

Zebrafish *med14* MO	ACTGGGAGATAAATCACATACCGCA
Zebrafish *smarcal1 MO*	GCTGAGTCTGTAAAGATGAGCATAA
Zebrafish *med14* RT-PCR-Fwd	GATGAAATCGCTTCCGCTG
Zebrafish *med14* RT-PCR-Rev	TTGACTCGTCCATTGGCCAC
Zebrafish *smarcal1* RT-PCR-Fwd	TTGTGTCAGTAAGCGCCTGT
Zebrafish *smarcal1* RT-PCR-Rev	CATCCCTTCCAGAGGTTTGA
Zebrafish *actb2* RT-PCR-Fwd	TGGCATCACACCTTCTAC
Zebrafish *actb2* RT-PCR-Rev	AGACCATCACCAGAGTCC
Mouse *Med14* V769A sgRNA	UUGAAAUGUUUCUUAATGAC
Mouse V769A mutant sgRNA binding site HDR donor oligo1	ACCATCCCGACATGTTTACCTGACGTATG AAAATTTGTTGTCTGAACCTGTTGGTGGC AGAAAAGTAGCTGAGATGTTCTTGAACGA TTGGAGTAGCATTGCCCGTTTATACGAGTG TGTGTTGGAATTTGCACGTTCTCTACCAGgta CACTTGGGTGGCTGAATTAG
Mouse V769A genotyping	GAGAAAGAGAGACTATACACTGCGG
Mouse V769A genotyping	TGTTCTGGTCATTGGCAGCCTGG
Human MED14 PCR-Rev	AAAGGAGATTATCTCCACACGTAC
Human MED14 PCR-Fwd	GTATAACTGAGGAAACCCAAAAGG
Human L1CAM PCR-Rev	TCTGAGTTGCATCTGAGGGTAA
Human L1CAM PCR-Fwd	TTCAGTGGTGAGTGTCTCGTC
Mouse *L1cam* Q497H sgRNA	GCCAATGGAACGCTGAGCATCAGAGACCTC CAGGCCAA
Mouse *L1cam* Q497H Donor Oligo	TGACACTGGACGCTATTTCTGCCAGGCCGCA AACGATCACAACAATGTGACCATTTTGGCTA ACCTACAGGTTAAAGGTTAGATGATGAGCAC ACATGACTG
Mouse *L1cam* Q497H PCR-Rev	ATCTCCACGCCAAGTGATGCT
Mouse *L1cam* Q497H PCR-Fwd	AGTGGTGAGTGCCCATC

### Construction of knockin mice

*Med14^V769A^* knockin mice were generated by the Fox Chase Transgenic Mouse Facility using CRISPR-induced cutting and HDR repair (33). *Med14^V769A^* mice were created using a single guide RNA (sgRNA) close to the mutation site (all oligonucleotides are listed in **Table 1**) and a 150 bp oligonucleotide donor encoding the T to C change plus 5 silent mutations in the sgRNA binding site to prevent cutting of the altered allele. The knockin mutation created a new Alu I restriction enzyme site which was used for screening for the allele. The *L1cam*^Q497H^ mouse line was also generated using the CRISPR/Cas9 method. Briefly, an sgRNA (**Table 1**) designed to cut near the *L1cam* mutation site was purchased from Synthego (Menlo Park, CA). Cas9 mRNA was purchased from TriLink BioTechnologies (San Diego, CA). Single strand donor oligonucleotide for *L1cam* (**Table 1**) was used to introduce point mutations (IDT, Coralville, IA). Besides the desired nucleotide changes to convert the Q to H, four silent nucleotide substitutions that prevented Cas9 from continuously cutting the DNA after donor knock-in were also included in the donor oligonucleotides. For making the *L1cam* knockin mouse line, the sgRNA (20 ng/μl) and its corresponding donor oligonucleotides (100 ng/μl) were co-microinjected with Cas9 mRNA (50 ng/μl) into the cytoplasm of zygotes from C57BL/6 mice (Charles River Laboratory) and the resulting embryos were implanted into the oviducts of pseudo-pregnant surrogate mothers. Offspring born to the foster mothers were genotyped by PCR and Sanger DNA sequencing and founders with the desired nucleotide changes were identified. Founder mice were backcrossed to C57BL/6J (JAX 000664) background for 4-6 generations before using for experiments.

### Flow cytometry

Single-cell suspensions from thymus and spleen were stained, as indicated, with optimal amounts of the following fluorochrome-conjugated antibodies: anti-CD3ϵ (145-2C11), anti-CD4 (GK1.5), anti-CD8 (53-6.7), anti-CD24 (M1/69), anti-CD25 (PC61), anti-CD44 (IM7), anti-CD62L (MEL-14), anti-CD69 (H1.2F3), anti-CD73 (TY/11.8), anti-CD90.2 (30-H12) anti-B220 (RA3-6B2), anti-NK1.1 (PK136), anti-CD122 (TM-β1), anti-TCRδ (GL3), anti-TCRβ (H57-597), anti-IgM (RMM-1) and CFSE Cell Division Tracker Kit 423801. The antibodies were purchased from BD Biosciences, eBioscience, BioLegend, or Tonbo Biosciences. Dead cells were excluded from analyses using propidium iodide (PI). Data were acquired on an LSRII flow cytometer (BD Pharmigen) or FACS Canto II flow cytometer (BD Pharmingen) and analyzed with Flowjo 9.96 software (Treestar, Inc.). CSFE dilution was employed to monitor proliferation of splenic T cells from WT or *L1cam*^Q497H^ mutant mice according to manufacturers specifications (CellTracer CFSE Cell Proliferation Kit, Invitrogen, Carlsbad, CA) following stimulation with 2 μg/ml plate-bound anti-CD3 and 1 μg/ml soluble anti-CD28.

### Graphing and statistics

Graphical analysis was conducted using GraphPad prism V9 and statistical significance was calculated using one-way ANOVA and student t tests. Significance values are indicated.

## Results

### Identification of a proband with T lymphopenia

The male proband was born at term to nonconsanguineous, healthy parents who had no known family history of immunodeficiency, as described (patient #5) (17). Newborn SCID screening was positive with a TREC level of 3, and confirmatory testing showed T-cell lymphopenia with 1,021 T cells/μl, essentially absent naïve T cells, and normal B and NK cell numbers (**Table 2**). The patient had a non-dysmorphic appearance, had no syndromic features, and negative testing included FISH for 22q11.2 microdeletion, ADA/PNP metabolites, and sequencing a panel of previously reported SCID genes. Imaging analysis at age 6 months, 15 months, and 4 years detected tissue in the anatomic location of the thymus, but it was diminished in size and displayed fatty infiltration. T cell proliferation to mitogen was normal, but was severely impaired following TCR cross-linking. Testing for maternal engraftment was negative. Thus, the immune phenotype was leaky/atypical SCID per Primary Immune Deficiency Treatment Consortium (PIDTC) criteria (35). The patient was closely monitored in protective isolation, maternal breast milk was discontinued, and IVIG and *Pneumocystis jirovecci* prophylaxis were given. Low-level CMV was detected by PCR in the patient’s blood at 10 months of age. The patient was asymptomatic, but was subsequently treated with valganciclovir for persistent low-level CMV viremia. At 14 months of age, EBV was detected by PCR in the patient’s blood, again without signs or symptoms for EBV infection. Because of the leaky/atypical SCID phenotype and persistent CMV and EBV, an allogeneic conditioned hematopoietic stem cell transplant (HSCT) was performed at 15 months of age using the patient’s healthy HLA-matched older brother as the donor. The brother was in good health, had passed the newborn SCID screen, and normal extended immune phenotyping, normal quantitative immunoglobulins and robust vaccine titers. The conditioned HSCT was uncomplicated, with both T and myeloid donor engraftment (81% and >95% respectively at Day +60) and undetectable levels of EBV and CMV at Day +60 after HSCT. The patient’s T cell functional studies normalized; however, his T lymphopenia did not improve over time (**Table 2**). The patient remains alive and well off all immune supportive therapies.

**Table 2:** Patient Immune Characteristics.

AGE	Days post-HSCT	Abs. CD3 num./µL(Ref. range)	Abs. CD4 T cell num./µL(Ref. range)	Abs. CD8 T cell num./µL(Ref. range)	Abs. B cell (CD19+) num./µL(Ref. range)	Abs. NK cell num. (CD3-CD16+/CD56+)/µL(Ref. range)	%CD3+CD4+CD45RA+(Ref. range)	TREC level*
1 week	n/a	1021 (2500-5500)	756 (1600-4000)	265 (560-1700)	1777 (300-2000)	869 (170-1100)	2 (64-95)	3
1 month	n/a	702 (2500-5500)	488 (1600-4000)	214 (560-1700)	1922 (300-2000)	397 (170-1100)	2 (64-95)	0
3 months	n/a	834 (2500-5500)	516 (1600-4000)	278 (560-1700)	2184 (300-2000)	953 (170-1100)	3 (64-95)	n.d.
6 months	n/a	497 (1900-5900)	276 (1400-4300)	221 (500-1700)	1586 (610-2600)	390 (160-950)	6 (64-93)	0
12 months	n/a	708 (2100-6200)	248 (1300-3400)	425 (620-2000)	1876 (720-2600)	920 (180-920)	6 (63-91)	0
21 months	6 months	662 (2100-6200)	95 (1300-3400)	93 (620-2000)	761 (720-2600)	683 (180-920)	0 (63-91)	n.d.
27 months	1 year	529 (1400-3700)	88 (700-2200)	382 (490-1300)	764 (390-1400)	176 (130-720)	1 (53-86)	n.d.
39 months	2 years	347 (1400-3700)	95 (700-2200)	210 (490-1300)	599 (390-1400)	95 (130-720)	3 (53-86)	0
6 years	5 years	684 (1200-2600)	151 (650-1500)	456 (370-1100)	n.d	n.d.	10 (46-77)	0
10 years	9 years	750 (1200-2600)	392 (650-1500)	303 (370-1100)	n.d.	n.d.	6 (46-77)	98 (≥5270)

TREC level assayed by two methods: through newborn screening laboratory with blood filter card and liquid blood sample through CLIA laboratory,

n/a, not applicable.

n.d., not done.

Reference ranges (34).

### Identification of candidate variants by exome sequencing

**Figure 1:**
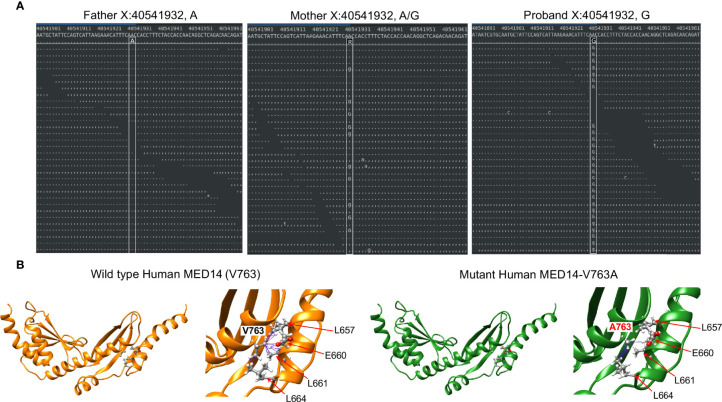
Identification of the *MED14* missense mutation by next generation sequencing. **(A)** Screen shots of NGS sequencing runs of the proband and parents are depicted. The A>G mutation is indicated by upper and lower case G. Dots or commas indicate wild type sequence. **(B)** Molecular models of the wild type and variant MED14 proteins. Two views of wild type (orange) and V763A mutant (green) human MED14 are depicted. The right half of each panel shows a zoomed in view of aa 763 with nearby residues on the opposing helix that are capable of making contacts with the A or V763. The left panel shows wild type MED14 V763 from known PDB structure 7ENA chain n, residues 637 to 884. The right panel shows the human V763A MED14 variant. Hydrophobic contacts are shown with purple lines.

To determine the genetic cause for the patient’s disease, WES was performed on genomic DNA from the proband and his parents as described (7). The variants prioritized by our initial analysis were an X-linked V763A variant in *MED14* and a homozygous autosomal R114H variant in *SMARCAL1* (**Figure 1A** and **Supplementary Figure 1A**). SMARCAL1/HARP is an annealing helicase that functions in the repair and restart of damaged DNA replication forks and has been linked to AR Schimke immuno-osseous dysplasia (SIOD), which can cause T-cell immunodeficiency, but is also accompanied by short stature and other phenotypes (36). The affected arginine residue (R114) in SMARCAL1 was not conserved between human, mouse, and zebrafish (**Supplementary Figure 1A**) and its location in the SMARCAL1 protein was distinct from that of reported pathogenic mutations that cause SIOD (37, 38).

### Functional testing of candidate variants

To test in zebrafish the role of the *smarcal1* gene in supporting T cell development, expression of *smarcal1* was knocked down using MO that disrupted pre-mRNA splicing, following which the impact on T cell development was evaluated by WISH using an *lck* probe to identify T cells (**Supplementary Figure 1B**). Importantly, despite effective induction of *smarcal1* mis-splicing, the development of T cells at 5 dpf was not impaired, suggesting that *smarcal1* was not essential for T cell development in zebrafish and that the SMARCAL1 R114H variant was unlikely to be responsible for the T lymphopenia observed in the proband.

The other highly ranked variant was in MED14, an integral component of the mediator complex that links the head and neck of the complex (39). While mediator complex component MED23 has been implicated in T cell activation, mediator complex function has not been explored in T cell development (40). The MED14 V763 residue that was mutated in the proband was conserved from human to zebrafish (**Supplementary Figure 2A**). To explore the extent to which the V763A variant might damage MED14 function, we performed structural modeling using Rosetta to optimize side chain packing and obtain an energy score reflective of the stability of the V763A variant compared to wildtype MED14 (23, 25). The results showed that the V763A substitution resulted in a decrease in hydrophobic contacts between two key helices at the ‘elbow’ of MED14 between repetitive modules (RM) 5 and 6 (**Figure 1B**). V763 made 16 contacts to 4 different residues (L657, E660, L661, L664), while the A763 variant made only 5 contacts to 2 residues (E660, L664), decreasing the stability of the A763 variant by 7.9 Rosetta Energy Units (REU) (26), far more profoundly than V to A substitutions in 11 other model proteins, which averaged reductions of 2.4 kcal/mole (41). It was not clear whether the reduction in hydrophobic contacts observed in the V763A variant was sufficient to cause major changes in MED14 conformation, but it was likely to cause local structure perturbation that could affect protein turnover and/or alter conformational dynamics.

**Figure 2:**
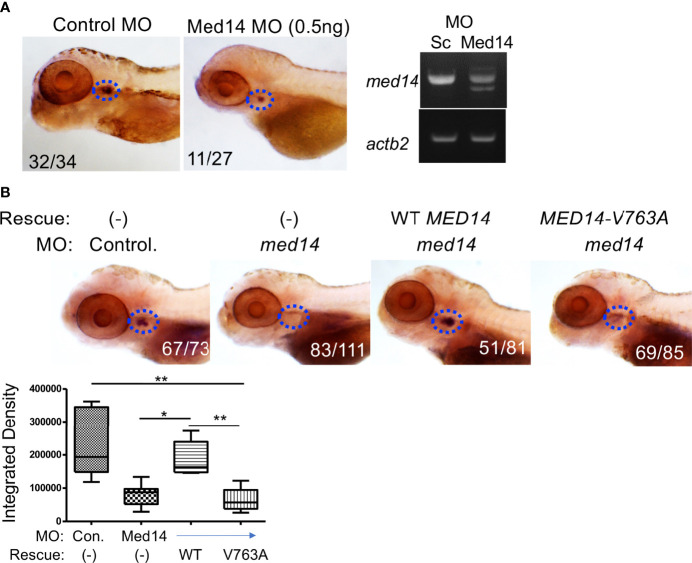
Role of Med14 in Zebrafish T cell Development. **(A)** The effect of MO knockdown of *med14* on T cell development at 5 dpf was assessed by WISH using an *lck* probe to identify T cells. The numbers on the images reflect the fraction of the embryos with the depicted staining pattern. Thymus staining is outlined by blue dashed ovals. The panel on the right confirms MO induced mis-splicing of *med14* mRNA by RT-PCR at 1 dpf with β-actin (*actb2)* as a loading control. **(B)** The ability of the wild type and human MED14 variant to rescue loss of endogenous zebrafish *med14* was assessed by heat-inducible re-expression of wild type or variant MED14. The effect on T cell development was assessed as above by WISH using an *lck* probe. The integrated density of WISH staining was measured by ImageJ software and depicted graphically as box plots. Significantly altered groups are indicated. Data are representative of 3 experiments. * p < 0.05, ** p < 0.01.

To investigate the role of Med14 protein in supporting T-cell development *in vivo*, we performed MO knockdown of *med14* in zebrafish. The role of zebrafish *med14* in T cell development had not previously been evaluated because the zebrafish *logelei* mutant (in *med14)* arrests embryo development at 2 dpf (15). Knockdown of *med14* using splice-site blocking MO at the indicated dose disrupted the splicing of *med14* pre-mRNA, without generally disrupting zebrafish development or altering morphology; however, WISH using an *lck* probe to identify T cells in the thymus at 5 dpf revealed that *med14* knockdown markedly impaired T cell development (**Figure 2A** and **Supplementary Figure 2B**). The decrease in Lck^+^ T cells indicated that Med14 plays a critical role in supporting T cell development in zebrafish. To determine whether the specific V763A patient variant (**Figure 1A** and **Supplementary Figure 2A**) would impair MED14 function, as predicted by structural modeling (**Figure 1B**), we performed rescue experiments. After knocking down endogenous *med14* expression using splice blocking MO, wild type or patient variant human *MED14* were re-expressed using heat-mediated induction (7). Expression of the human wildtype MED14 protein rescued the arrest in T cell development caused by knockdown of endogenous *med14*, indicating conservation of function between zebrafish and human MED14 (**Figure 2B** and **Supplementary Figure 2C**). Importantly, however, re-expression of the patient *MED14^V763A^* variant failed to rescue the loss of endogenous *med14* (**Figure 2B**), indicating that the *MED14^V763A^* variant significantly damaged MED14 function. These findings suggested that the *MED14^V763A^* variant might have caused the proband’s immunodeficiency.

### Basis for impaired T cell development upon Med14 loss

**Figure 3:**
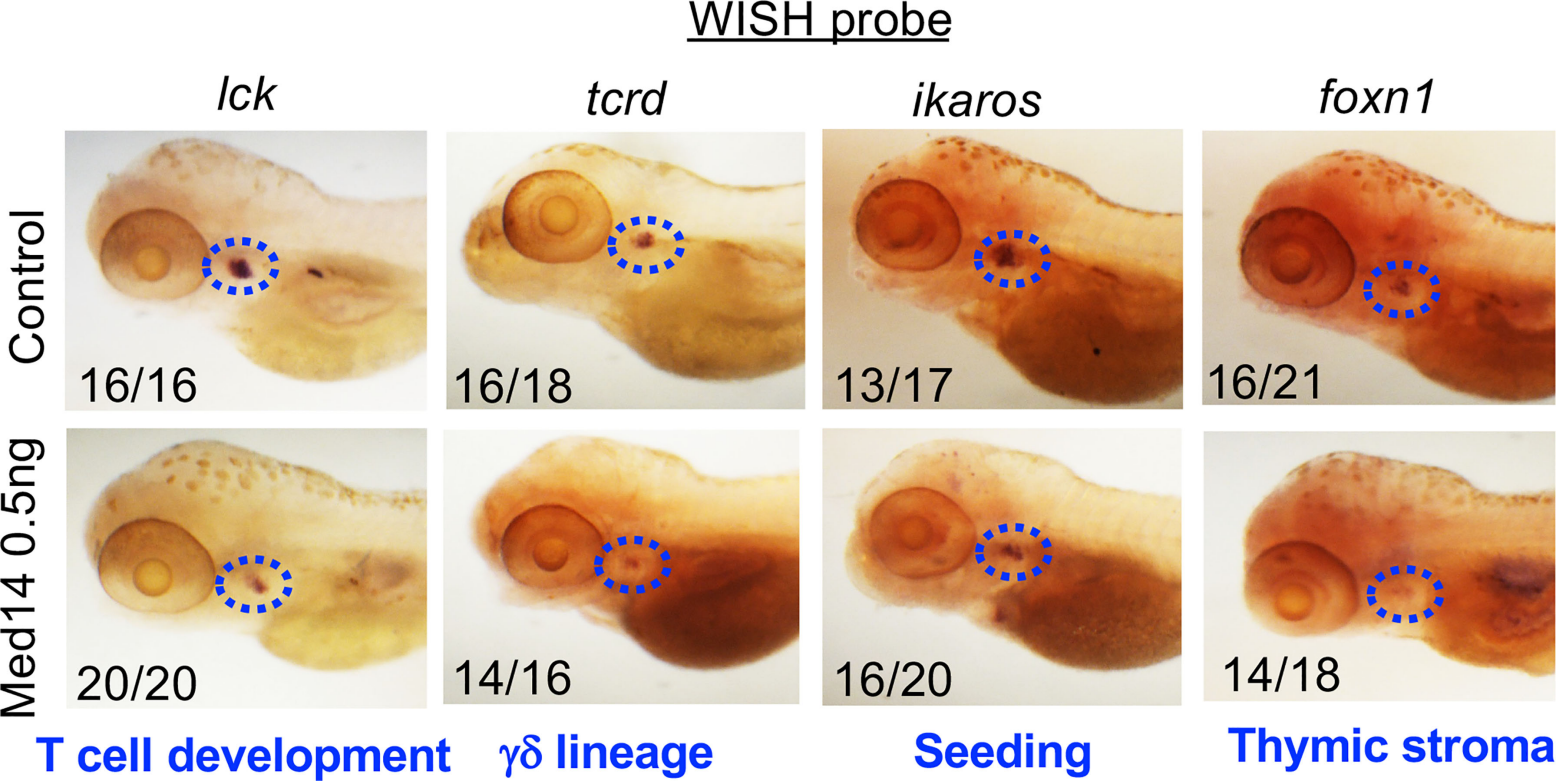
Role of MED14 in Development of Thymic Subpopulations in Zebrafish. The effect of *med14* knockdown on cell subpopulations was evaluated at 5 dpf by performing WISH on TLF zebrafish embryos with the indicated probes: *lck* marks most developing thymocytes, *ikaros* marks thymic seeding progenitors, *tcrd* marks γδ lineage progenitors, and *foxn1* marks thymic epithelial cells. Blue ovals mark the thymus and frequencies of embryos with the exhibited staining pattern are indicated at the lower left of each image. Data are representative of 3 experiments.

To determine how Med14 loss blocked zebrafish T cell development, we examined whether other precursor and cell populations were impacted. Because *lck* is expressed in all T cell precursors, the reduction in the *lck* WISH signal (**Figure 3**) indicated that overall thymic cellularity was reduced in zebrafish in the absence of Med14. To determine if the impairment of T cell development was restricted to the αβ T cell lineage or affected both αβ and γδ T lineage cells, we performed WISH with a probe for *tcrd*, which marks γδ T lineage cells (**Figure 3**). The reduction of both *lck*- and *tcrd-*marked T lineage precursors indicated that Med14 loss impaired the development of both the αβ and γδ T cell lineages (**Figure 3**). WISH employing a probe for *ikaros* to mark thymic seeding cells revealed that the loss of Med14 reduced thymic seeding (**Figure 3**). Finally, WISH using *foxn1*, which marks thymic stroma, revealed reduced staining, suggesting that the thymic structure itself might also have been disrupted (**Figure 3**). Importantly, similar results were obtained when the zebrafish *med14* knockdown was replaced with the *MED14^V763A^* variant, indicating that the patient variant was unable to rescue the attenuation of thymic seeding or perturbation of thymic stroma (**Supplementary Figure 3**). Taken together, these observations indicated that loss of Med14 function interfered with development by attenuating thymic seeding and might involve impaired thymic organogenesis.

### Effect of the *MED14^V763A^* mutation on T cell development in mice

**Figure 4:**
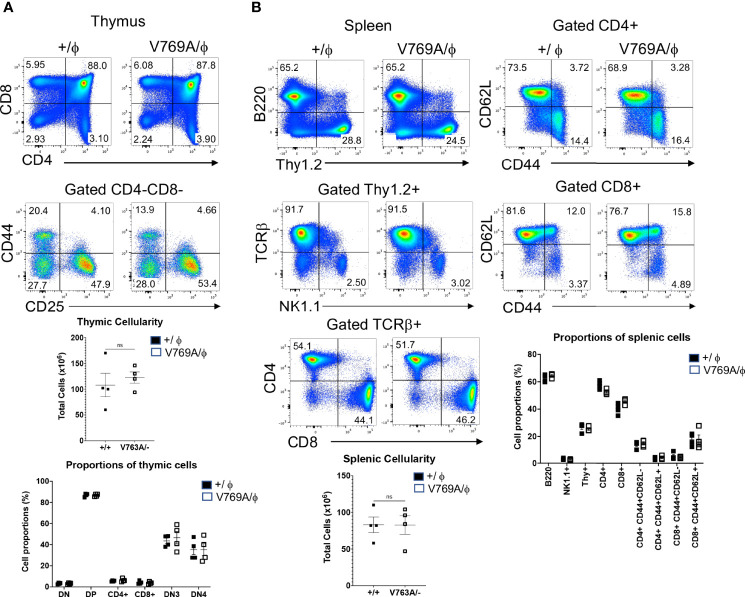
Phenotypic analysis of lymphoid development in *Med14* mutant mice. **(A)** Histograms are displayed of flow cytometry analysis of thymic cell suspensions from wildtype (+/ϕ) and hemizygous *Med14* mutant (V769A/ϕ) mice. The following antibodies were used: CD4, CD8, CD44, and CD25. Scatter plots of total thymic cellularity and the frequencies of the indicated populations are depicted. The following populations are graphed: DN, CD4-CD8-; DP, CD4+CD8+; CD4+; CD8+; DN3, CD4-CD8-CD44-CD25+; DN4, CD4-CD8-CD44-CD25-. Proportion of DN3 and DN4 subpopulations among DN thymocytes is depicted graphically **(B)** Histograms are displayed that illustrate flow cytometric analysis of the lymphoid content of spleens from +/ϕ and V769A/ϕ mice. The following antibodies were used: B220, Thy1.2, NK1.1, CD4, CD8, TCRβ, CD44, and CD62L. Scatter plots of total splenic cellularity and the frequencies of the indicated populations are depicted. Each symbol represents an individual mouse. The proportions of CD4 and CD8 T cells among Thy1+ cells and the proportions of memory subsets among CD4+ and CD8+ subsets are depicted graphically. Data are representative of 3 experiments performed. No statistically significant differences were found in any of the indicated populations.

Because the zebrafish model did not allow one to ascertain the precise stage of developmental arrest upon Med14 loss or whether it was cell-autonomous, we next developed a mouse model in which these questions could be addressed by generating V769A knock-in mice *(Med14^V769A^)* (**Supplementary Figure 4**). These mice were viable and fertile and were analyzed after being outcrossed to C57BL/6 mice for at least 4 generations. To determine if the *Med14^V769A^* variant knock-in mice exhibited a defect in T cell development, we performed flow cytometry on thymic and splenic explants (**Figure 4**). Surprisingly, analysis of the thymus revealed no reduction in cellularity in the *Med14^V769A^* knockin mice or in the proportion of thymic subsets (**Figure 4A**). Moreover, there was no change in splenic cellularity or the distribution of B, T, or NK cells in the spleen, including in memory T cell subsets defined by CD44 and CD62L (**Figure 4B**). However, competitive transplantation analysis revealed that hematopoietic stem and progenitor cells (HSPC) from *Med14^V769A^* mice exhibited a mild impairment of differentiation beyond the β-selection checkpoint as evidenced by an accumulation of CD4^-^CD8^-^CD44^-^CD25^+^ (DN3) thymocytes, reduced DN3b (CD25+CD98+) and DN4 (CD44-CD25-), and strongly reduced T cell repopulation of the periphery (**Supplementary Figure 5**). The reduction of peripheral T cells was not associated with decreases in thymic emigrating cells (CD69-CD62L+S1PR1+) or reduced proliferation in the periphery (**Supplementary Figure 5**).

The absence of a baseline defect in T cell development in the *Med14^V769A^* mice prompted us to investigate whether the V to A substitutions impacted the structure of mouse and human MED14 protein differently. Consequently, we replicated the structural modeling analysis that we had performed on the human V763A variant (**Figure 1B**) using ColabFold (27). The resulting AlphaFold 2 model had a complete chain structure that was highly similar to the conformation of the PDB 6W1S mouse MED14, with a root-mean-square deviation (RMSD) of 160 alpha carbons of 0.983 Å, and an overall RMSD of 228 protein structure pairs of 1.531 Å (**Supplementary Figure 6**). The human and mouse sequences were highly conserved in this region of MED14, with 98% identity. Only 4 positions in the aligned fragment (residues 643 to 890) differed. Nevertheless, the same coordinate-constrained Rosetta relaxation protocol (25, 26) demonstrated that the murine V769A variant exhibited only a minor reduction in predicted stability of 1.2 REUs relative to the wildtype protein (**Supplementary Figure 6**). The difference relative to human MED14 V763 was that murine MED14 V769 made 17 hydrophobic contacts (relative to 7 in the human MED14 V763) with 4 different residues (L663, E666, L667, L670) on the adjacent helix (**Supplementary Figure 6**). This increase in contacts apparently rendered mouse MED14 resistant to the A substitution, consistent with a possible difference in mouse versus human MED14 proteins harboring the V763A change.

### Sequence analysis of the unaffected sibling

**Figure 5:**
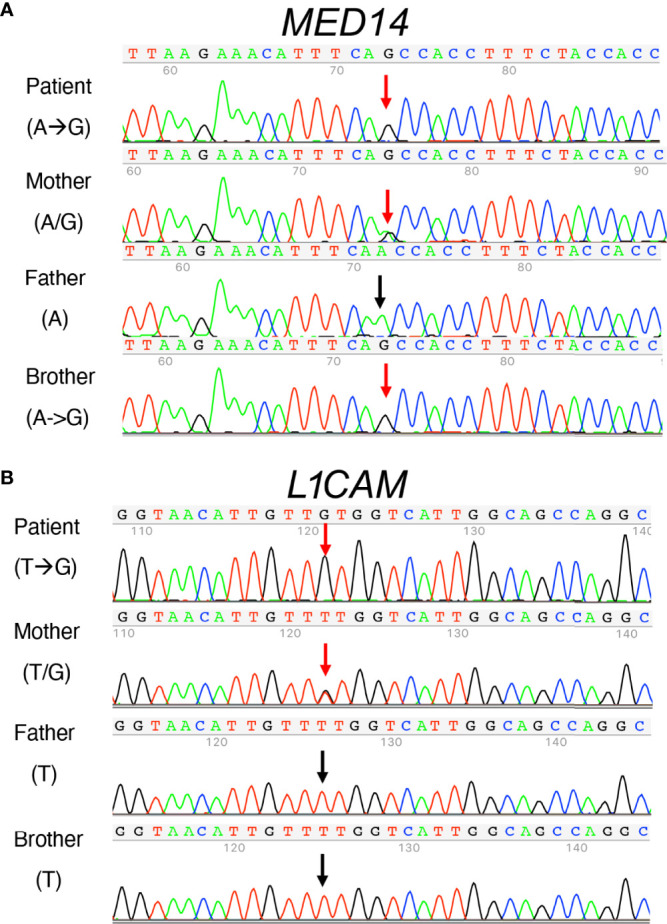
Sanger sequence analysis of MED14 V763A and L1CAM Q498H variants in the patient, his parents, and his healthy brother. **(A)** The mother carries both alleles with A and G The patient and his healthy brother inherited the same alleles with G, resulting in the same MED14 V763A variant as indicated by red arrows. The PCR-Rev primer was used for sequencing. **(B)** Patient’s mother carries T and G alleles (red arrow). The patient inherited the allele with G (red arrow), resulting in the L1CAM^Q498H^ variant and his healthy brother inherited the allele with T (see black arrow). His father’s allele also has a T (black arrow). PCR-Fwd primer was used for sequencing.

**Figure 6:**
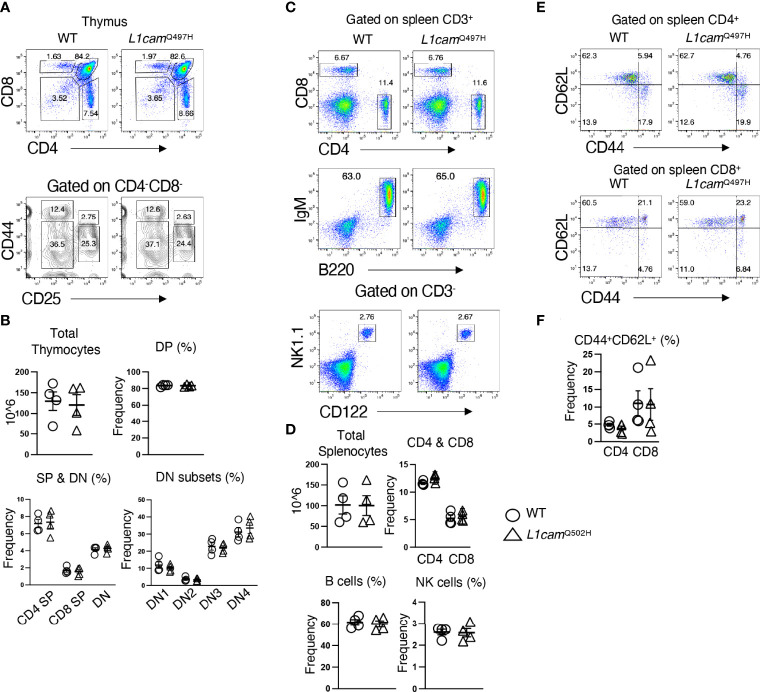
Phenotypic analysis of the *L1CAM^Q497H^* knockin mice. **(A)** Flow cytometric analysis of the frequencies of thymocytes from wildtype (WT) and hemizygous *L1cam^497H^* mutant mice. **(B)** The total and subpopulations of thymic cellularity are shown in bar graphs. The following populations are graphed: DN, CD4^-^CD8^-^; DP, CD4^+^CD8^+^; CD4^+^; CD8^+^; DN1, CD4^-^CD8^-^CD25^-^CD44^+^; DN2, CD4^-^CD8^-^CD25^+^CD44^+^; DN3, CD4^-^CD8^-^CD44^-^CD25^+^; DN4, CD4^-^CD8^-^CD44^-^CD25^-^. **(C)** Flow cytometry analysis of splenic CD4^+^ and CD8^+^ T cells, B (B220^+^IgM^+^) and NK (CD3^-^CD122^+^NK1.1^+^) cells in WT and *L1cam*^Q497H^ mutant mice. **(D)** The total and subpopulations of splenic cellularity are shown in bar graphs. **(E)** Flow cytometry analysis of CD44 and CD62L staining of CD4^+^ and CD8^+^ T cells. **(F)** The frequency of CD44^+^CD62L^+^ in CD4^+^ and CD8^+^ T cells is shown in bar graphs. Data are representative of 2 independent experiments performed. No statistically significant differences were found in any of the indicated populations.

The inability of the murine *Med14*^V769A^ variant to attenuate baseline T cell development diminished the likelihood that this variant alone could fully account for the immunodeficiency in the patient. To seek other potentially disease-causing candidate gene(s), WES was repeated using the patient, his parents, and his healthy brother, the HSCT donor. Unexpectedly, the brother shared the same *Med14*^V763A^ variant as the patient (**Figure 5A**), indicating that this genotype alone could not explain the disease. This raised the possibility that there was variable penetrance of a phenotype due variable expressivity, in which identical mutations may be associated with a spectrum of disease severity due to the contributions of secondary mutations that differ between patients (42–44). Another possibility was that a gene other than *MED14* could be responsible for the disease (see Discussion). Additional variants were indeed identified in 6 genes (**Table 3**), including an X-linked variant in *L1CAM* (p.Q498H) present in the patient but not in his healthy brother (**Figure 5B**). L1CAM is a transmembrane glycoprotein belonging to the immunoglobulin superfamily of cell adhesion molecules (45). It contains six immunoglobulin (Ig) and five fibronectin III-like domains at the extracellular surface, a single-pass transmembrane domain, and a short cytoplasmic domain (46). The Q498H variant was located in the fifth Ig domain. Mutation or deletion of L1CAM has been associated with an X-linked recessive neurological disorder (47), with at least 248 variants/mutations having been identified (48), but the roles of the variants, including the one in the proband, have not been studied in the immune system. To further examine whether an orthologous murine *L1CAM*^Q497H^ variant (corresponding to *L1CAM*^Q498H^ in human) would result in T cell deficiency, we generated *L1cam*^Q497H^ knock-in mice (**Supplementary Figures 7A, B**). However, no defects were observed, with normal development of CD4^+^CD8^+^ (DP), CD4^+^ and CD8^+^ (SP), CD4^-^CD8^-^ (DN), DN1 (CD25^-^CD44^+^), DN2 (CD25^+^CD44^+^), DN3, and DN4 (CD25^-^CD44^-^) cells in thymus (**Figures 6A**, **B**); normal overall frequencies and numbers of T, B (B220^+^IgM^+^) and NK (CD3^-^CD122^+^NK1.1^+^) cells in spleen (**Figures 6C**, **D**); and normal memory T cell subsets (CD44^+^CD62L^-^) (**Figures 6E**, **F**). In addition, the proliferative response to anti-CD3 plus anti-CD28 was also normal (**Supplementary Figure 7C**). These data showed that L1CAM^Q497H^ in the mouse was not sufficient to cause a defect in the development of T cells.

**Table 3:** List of potentially interesting variants identified by whole exome-sequencing.

Chr	Start	End	Ref	Alt	Gene	Location	Domain	snp138	D	ConsSites	Model	Father	Mother	Child	Brother
chrX	153134053	153134053	T	G	L1CAM	Exon11-:p.Q498H	C2	na	15.16	SOX9_B1	X-link	0%	46%	100%	0%
chr14	103571109	103571109	T	C	EXOC3L4	Exon5-:p.I440T	Sec6	na	19.66	1	AR	53%	52%	100%	50%
chr14	104436947	104436947	C	T	TDRD9	Exon6:p.R279C	DEXDc	na	20.2	na	AR	55%	64%	100%	27%
chr8	38091971	38091971	G	T	DDHD2	Exon3:p.G94W	WWE	rs202216406	17.17	na	AR	37%	58%	100%	58%
chr9	113132258	113132258	C	A	SVEP1	Exon47:p.V3547L	–	rs192794123	12.6	na	AR	52%	57%	100%	0%
chr1	55247289	55247289	C	T	TTC22	Exon7:p.G446D	–	na	27.5	na	Denovo	0%	0%	47%	0%
chrX	40541932	40541932	A	G	MED14	Exon18:p.V763A	–	na	22.5	EN1_01	X-link	0%	45%	100%	100%

Shown are either X-linked, Denovo, or autosomal recessive (AR) variants. Chr (Chromosome), Start (chromosomal start), End (chromosomal end), Ref (Refseq), Alt (alteration), Gene (gene name), Location (chromosome location and corresponding protein sequence), snp138 (dbSNP buiding 138), CADD (combined annotation dependent depletion), ConsSites (target gene per GSEA database), Model (type of variant), and the percentage of variants in each individual are shown, with 100% being homozygous and 50% or less being heterozygous.

## Discussion

Here we describe a male proband identified through newborn screening who exhibited T-B+NK+ immunodeficiency. Informatic analysis of our initial WES suggested that a missense mutation in the X-linked *MED14* gene might be responsible for the disease. This possibility is supported by functional analysis in zebrafish. Nevertheless, subsequent introduction of the patient variant into a knock-in mouse did not replicate the baseline T cell developmental arrest observed in zebrafish, although HSPC from these mice failed to fully reconstitute peripheral T cells in the competitive transplant setting. These data were of interest because the healthy male sibling of the proband also carried the *MED14^V763A^* variant, and while he exhibited no signs of baseline disease, his HSPC transplanted into the patient failed to generate normal T cell numbers, leaving the patient profoundly T lymphopenic (**Table 2**). These findings could be consistent with the *MED14^V763A^* variant potentially contributing to T lymphopenia; however, because both siblings shared the *MED14^V763A^* variant, that variant alone was insufficient to explain the proband’s disease. Consequently, variable expressivity, mediated by additional genetic variants, was considered. A search for such variants resulted in identification of a variant in the *L1CAM* gene in the proband, but not his healthy sibling. Nevertheless, introduction of the *L1cam* variant in mice had no effect on T cell development. Taken together, these data raise at least two potential etiologies in this patient. First, a distinct, yet unknown and undetected, gene mutation was actually responsible for his T cell insufficiency. Alternatively, the *MED14^V763A^* variant could underlie the patient’s T cell insufficiency, but the developmental defect was not manifest in the sibling because of variable expressivity (43, 44, 49).

Our analysis in zebrafish supported the interpretation that the *MED14^V763A^* mutation damages MED14 function and prevents it from supporting the development of Lck-expressing T cell development in the thymus, most of which are αβ lineage (50). Nevertheless, baseline impairment of T cell development was not observed in mice harboring the equivalent mutation. Molecular modeling of the mouse *Med14^V769A^* mutation suggested it might be less destabilizing than the human orthologous variant, providing a potential explanation for why the mouse *Med14^V769A^* mutation does not phenocopy the baseline defect in T cell development observed in zebrafish. Importantly, the murine *Med14^V769A^* variant impaired the ability of HSPC to reconstitute peripheral T cells, indicating that this variant is important for supporting development or maintenance of T cells. This also provides a possible explanation for the failure of the healthy sibling’s bone marrow to fully restore T cell numbers upon transfer into the proband. The mechanistic basis by which MED14 supports the function of HSPC and their capacity to fully reconstitute peripheral T cells remains unclear.

MED14 is a component of the 26-subunit Mediator Complex, a transcriptional coactivator transmitting signals from transcription factors to Polymerase II (Pol II) (13, 18, 19, 51). MED14 serves as a crucial backbone of the Mediator Complex (52). Consequently, the MED14 variant might compromise the capacity of the Mediator Complex to cooperate with transcription factors and Pol II to coactivate transcription, as exemplified by its critical role in PPARγ-dependent and glucocorticoid receptor-dependent transactivation of targets (53, 54).

While the structural differences between mouse and human MED14 might provide an explanation for the absence of a phenotype in our mouse model, this does not explain why the patient’s sibling also bears the same *MED14^V763A^* variant and yet is healthy. One possibility is the phenomenon of variable expressivity, widely observed in genetic disorders in which distinct individuals with identical mutations manifest marked differences in disease severity, even in siblings reared in the same environment (55–57). The prevailing view is that variable expressivity occurs because different complements of modifier gene variants influence disease penetrance (49). For example, cartilage hair hypoplasia (CHH), caused by mutations in the *RMRP* gene, which encodes an untranslated multifunctional RNA gene product, can manifest immune phenotypes that range from no significant impairment to T cell deficient typical SCID (58). Importantly, this variability is even observed among patients with identical *RMRP* mutations, presumably due to differences in modifier genes (43, 55, 59–63). Likewise, differences in modifier genes might explain the distinct disease penetrance between this patient and his male sibling with the same *MED14^V763A^* variant, assuming it is indeed responsible for the disease. The seemingly healthy sibling may also be experiencing a mild functional deficit in hematopoiesis given that his bone marrow failed to correct the T cell insufficiency upon adoptive transfer into the patient. The background variants potentially responsible are not known. As noted, a patient L1CAM variant was tested, but did not impair T cell development in a mouse model. Modifier genes underlying variable expressivity may be exceedingly difficult to identify.

In summary, the current study used newborn screening coupled with functional testing in zebrafish and in mice. While the X-linked missense mutation in *MED14* remains a potential candidate for the disease-causing allele in this patient, presumably acting together with modifier gene variants through variable expressivity, the disease might alternatively be caused by defects in another, as yet undefined gene, and perhaps could be due to a mutation in a noncoding (e.g., promoter or enhancer) element affecting gene expression rather than in a coding region. While additional investigations are required to determine the basis for the proband’s disease, it is possible that further human cases of T lymphopenia associated with *MED14* variants could be found; such evidence from multiple affected individuals would provide strong supporting evidence for pathogenicity of this gene.

## Data availability statement

The WES data presented in the manuscript have been deposited in dbGaP under accession numbers phs002968.v1.p1 and phs002990.v1.p1.

## Ethics statement

The studies involving human participants were reviewed and approved by Research activities were performed with parental informed consent under protocols approved by the institutional review boards (IRBs) at the University of California, San Francisco and National Heart, Lung, and Blood Institute (NHLBI), National Institutes of Health (NIH), Bethesda, MD. Written informed consent to participate in this study was provided by the participants’ legal guardian/next of kin. The animal study was reviewed and approved by Animal housing and handling were all performed in accordance with the approved protocols from the Fox Chase Cancer Center Institutional Animal Care and Use Committee (IACUC). Likewise, mouse experiments were performed under the auspices of IACUC-approved animal protocols, and all mouse strains were housed in accredited facilities at either Fox Chase Cancer Center or NIH. All experiments using mice at NHLBI were performed using protocols approved by the NHLBI Animal Care and Use Committee and followed NIH guidelines for use of animals in intramural research.

## Author contributions

RSe, JXL, JMP, WJL, and DLW drafted the manuscript. WJL and DLW take the primary responsibility for this paper as the corresponding authors. All authors contributed to the article and approved the submitted version. JMP and CMS provided care for the patient and contributed clinical data. RSe, JXL, EM, SS and BT performed experiments. SR, MK, AS, US, MA, RMD, CL, RSr, and SEB performed data analysis.. All authors contributed to the article and approved the submitted version.

## Funding

This work was supported by the National Institutes of Health (NIH) grants P30CA006927, P01AI138962, and the Bishop Fund. W.J.L. is supported by the Division of Intramural Research, NHLBI. SEB received support from TCS to the University of California, Berkley. RLD was supported by NIH grant R35 GM122517.

## Acknowledgments

We thank the following Fox Chase Cancer Center core facilities for their vital service: Imaging, Molecular Modeling, Transgenic Mouse, and Laboratory Animal/Zebrafish. We thank the NHLBI Transgenic Core for generation of the L1cam mutant mouse line and the NHLBI Sequencing Core for whole exome sequencing. We thank Dr. Ning Du for help with some experiments and Mr. Tesfay Gebregiorgis, NHLBI for mouse colony maintenance.

## Conflict of interest

Authors US, RA, and RS (Srinivasan) are employed by TATA Consultancy Services. SEB was a principal investigator on a research service agreement between TCS and the University of California, Berkley.

The remaining authors declare that the research was conducted in the absence of any commercial or financial relationships that could be construed as a potential conflict of interest.

## Publisher’s note

All claims expressed in this article are solely those of the authors and do not necessarily represent those of their affiliated organizations, or those of the publisher, the editors and the reviewers. Any product that may be evaluated in this article, or claim that may be made by its manufacturer, is not guaranteed or endorsed by the publisher.

## Supplementary material

Supplementary Figure 1 *smarcal1* knockdown does not impair T cell development. **(A)** Multisequence alignment of *SMARCAL1* sequences with the patient variant (R114H) shown in a red box. **(B)** Upper panel, effect of *smarcal1* knockdown on T cell development as measured by WISH with an *lck* probe. Blue ovals mark the thymus and the numbers on the images indicate the frequencies of embryos with the depicted phenotype. Lower panel, efficacy of the *smarcal1* MO as indicated by mis-splicing of the *smarcal1* pre-mRNA by RT-PCR analysis. β-actin (*actb2)* serves as a loading control. Results are representative of at least 3 experiments.

Supplementary Figure 2 Conservation of the MED14 variant. **(A)** Multiple sequence alignment of human, mouse and zebrafish MED14. Valine 763 is boxed in red and conservation indicated (identical *, highly similar: similar.). **(B)** Schematic representation of the *med14* gene structure with the position of the Exon 3-Intron 3 MO and primers for RT-PCR analysis indicated. Blue arrows indicate primers and blue rectangle the morpholino binding site. **(C)** RT-PCR analysis of the efficacy of *med14* MO at 1 and 5 dpf of the rescue experiment. RT-PCR analysis of both *med14* and β-actin (*actb2)* are shown. Results are representative of at least 3 experiments.

Supplementary Figure 3 Effect on re-expression of the MED14 variant on generation of thymic subpopulations. A rescue experiments was performed as in **Figure 2**, and the resulting embryos analyzed by WISH with the indicated probes (*lck, ikaros, tcrd and foxn1*) to evaluate the ability of the MED14 variant to rescue γδ T cell development, thymic seeding, and thymic architecture. Blue ovals mark the thymus. Numbers on the figures represent the frequency of the depicted phenotype. Results are representative of at least 3 experiments.

Supplementary Figure 4 Generation and sequence validation of MED14 V769A knockin mice. **(A)** The sgRNA target sequence is shown with the sgRNA sequence indicated and the region it binds highlighted in yellow, mutations in the protospacer adjacent motif (PAM) to prevent re-cutting are colored red and newly created restriction enzyme screening site is indicated in green. The sequence trace files show PAM mutations, specific V769A mutation, and silent mutations in male founder. An Alu I digest is shown with cleaved bands indicating digestion of the mutant allele.

Supplementary Figure 5 Assessment of *Med14^V769A/ϕ^* hematopoietic progenitor function by competitive bone marrow transplantation. 100x10^5^ allotype marked wild type (CD45.1) and *Med14^V769A/ϕ^* mutant (CD45.2) lineage negative hematopoietic stem and progenitor cells (HSPC) were combined and transferred together into CD45.1 recipients that had been treated with 1100 rads (2x550r, 4h apart) 24h earlier. Recipient mice were placed on antibiotic-treated water (polymyxin B sulfate and neomycin) for 3 weeks and analyzed 6 weeks after transplantation. Single cell suspensions of thymus **(A)** and spleen **(B, C)** were stained with CD45.1 and CD45.2 antibodies to distinguish the genotypes of transferred HSPC and with the indicated lineage markers. Bromodeoxyuridine (BrdU) labeling was conducted by staining permeabilized cells after 24h of labeling. Gate frequencies were calculated and depicted as bar graphs of the mean +/- standard deviation. Statistical significance were determined using the t-test. P-values are indicated on the graphs. NS, not significant.

Supplementary Figure 6 Molecular modeling of the wild type and variant mouse MED14 proteins. Two views of wild type (orange) and V769A mutant (green) mouse MED14 are depicted. The right half of each panel shows a zoomed in view of aa 769 with nearby residues on the opposing helix that are capable of making contacts with the A or V769. The top panel shows wild type mouse MED14 V769 from known PDB structure 6W1S chain I, residues 643 to 890. The bottom panel shows the mouse V769A MED14 variant. Hydrophobic contacts are shown with purple lines.

Supplementary Figure 7 Construction and analysis of the the *L1CAM^Q497H^* knockin founder mice. **(A)** Part of human and mouse L1CAM amino acid sequences were aligned and Q498 in human and Q497 in mouse are highlighted and yellow. **(B)** The founder mouse was identified by PCR using tail gDNA and Sanger sequencing using PCR-Rev primer; black arrows indicate silent mutations introduced to prevent from subsequently cutting by Cas9, which do not change the amino acid and the red arrows indicate a G to C change to make Q to H mutation in mouse. **(C)** *In vitro* proliferation assays were performed using splenic T cells isolated from wild type littermates (WT) and L1cam^Q497H^ (KI) mice, which were labeled with CFSE, stimulated with 2 μg/ml of plate-bound anti-CD3 and 1 μg/ml of anti-CD28 as indicated. Cell proliferation was determined by CFSE dilution.
