# Supplementary figures and images for: Investigation of the causal etiology in a patient with T-B+NK+ immunodeficiency
Source: Front Immunol. 2022 Jul 29;13:928252. doi: 10.3389/fimmu.2022.928252 (PMC9372720; doi:10.3389/fimmu.2022.928252)

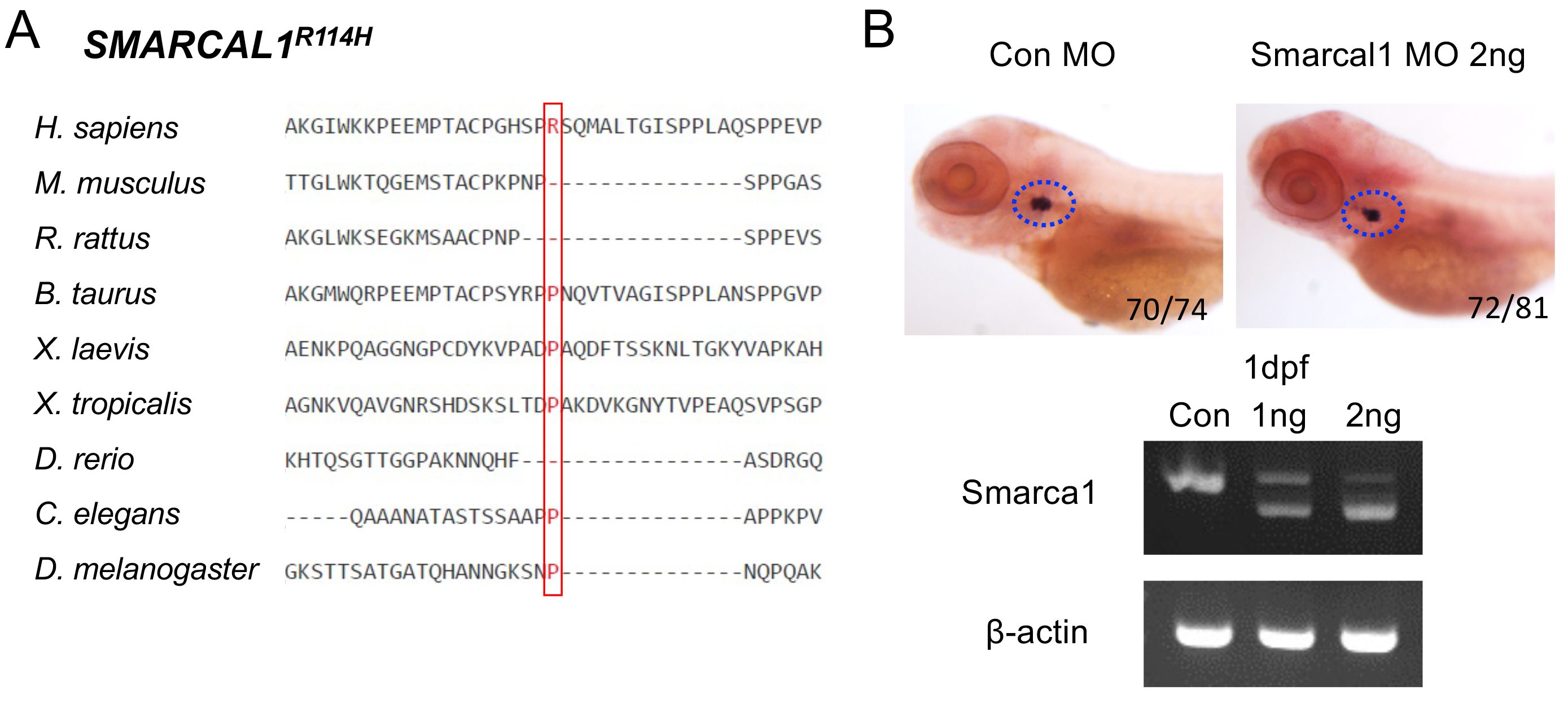

Supplement: Supplementary Figure 1 — smarcal1 knockdown does not impair T cell development. (A) Multisequence alignment of SMARCAL1 sequences with the patient variant (R114H) shown in a red box. (B) Upper panel, effect of smarcal1 knockdown on T cell development as measured by WISH with an lck probe. Blue ovals mark the thymus and the numbers on the images indicate the frequencies of embryos with the depicted phenotype. Lower panel, efficacy of the smarcal1 MO as indicated by mis-splicing of the smarcal1 pre-mRNA by RT-PCR analysis. β-actin (actb2) serves as a loading control. Results are representative of at least 3 experiments. [file Image_1.tif]

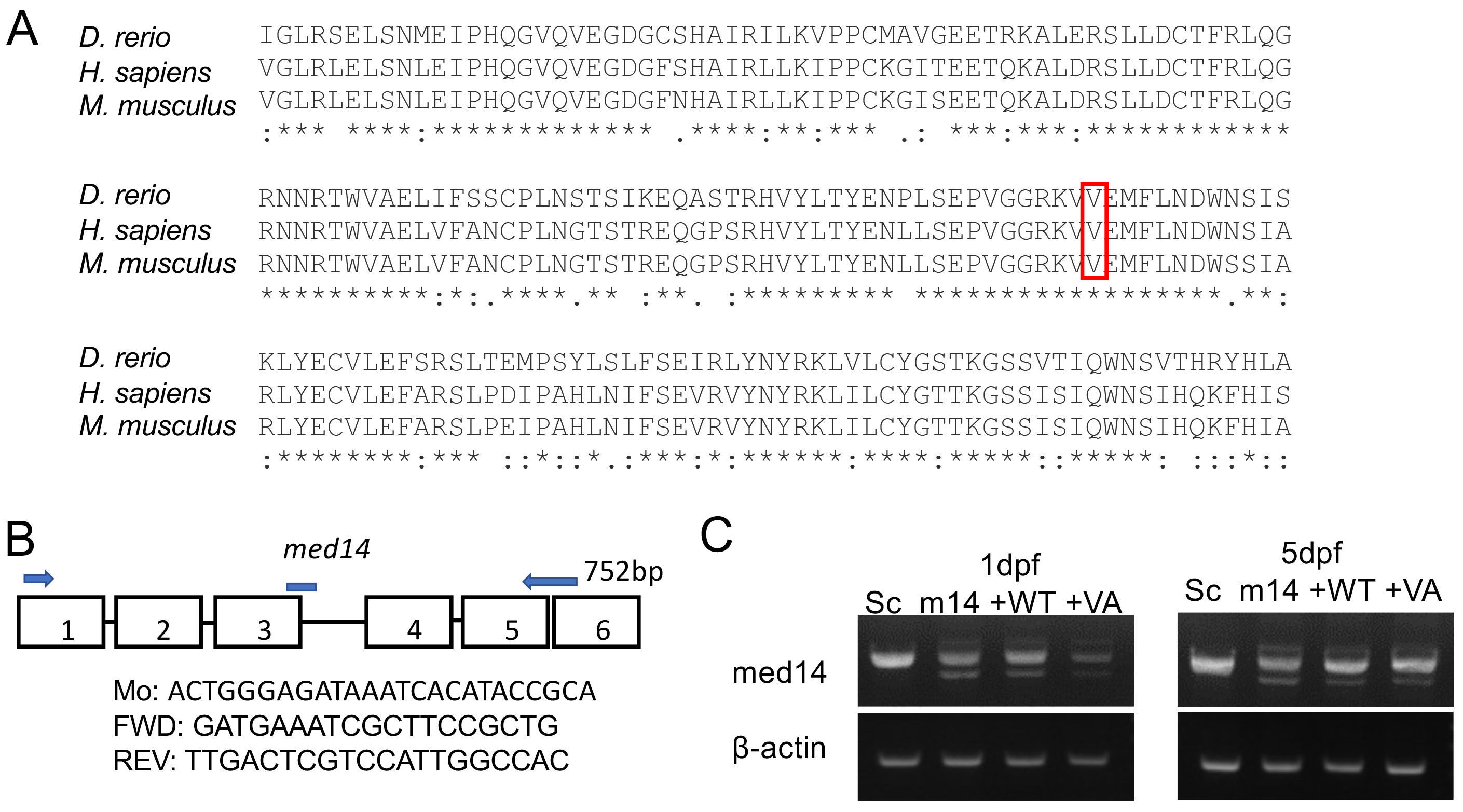

Supplement: Supplementary Figure 2 — Conservation of the MED14 variant. (A) Multiple sequence alignment of human, mouse and zebrafish MED14. Valine 763 is boxed in red and conservation indicated (identical *, highly similar: similar.). (B) Schematic representation of the med14 gene structure with the position of the Exon 3-Intron 3 MO and primers for RT-PCR analysis indicated. Blue arrows indicate primers and blue rectangle the morpholino binding site. (C) RT-PCR analysis of the efficacy of med14 MO at 1 and 5 dpf of the rescue experiment. RT-PCR analysis of both med14 and β-actin (actb2) are shown. Results are representative of at least 3 experiments. [file Image_2.tif]

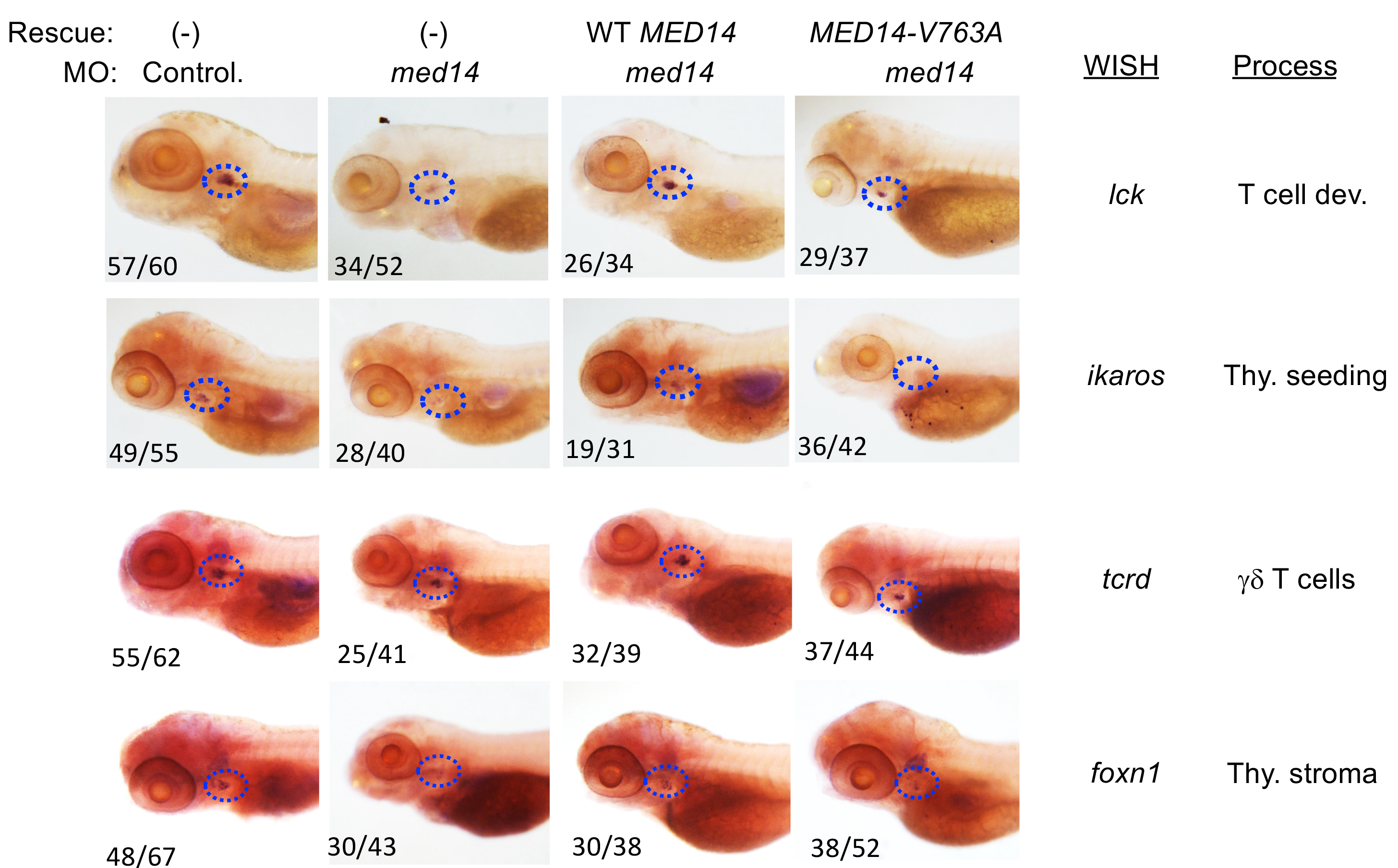

Supplement: Supplementary Figure 3 — Effect on re-expression of the MED14 variant on generation of thymic subpopulations. A rescue experiments was performed as in Figure 2, and the resulting embryos analyzed by WISH with the indicated probes (lck, ikaros, tcrd and foxn1) to evaluate the ability of the MED14 variant to rescue γδ T cell development, thymic seeding, and thymic architecture. Blue ovals mark the thymus. Numbers on the figures represent the frequency of the depicted phenotype. Results are representative of at least 3 experiments. [file Image_3.tif]

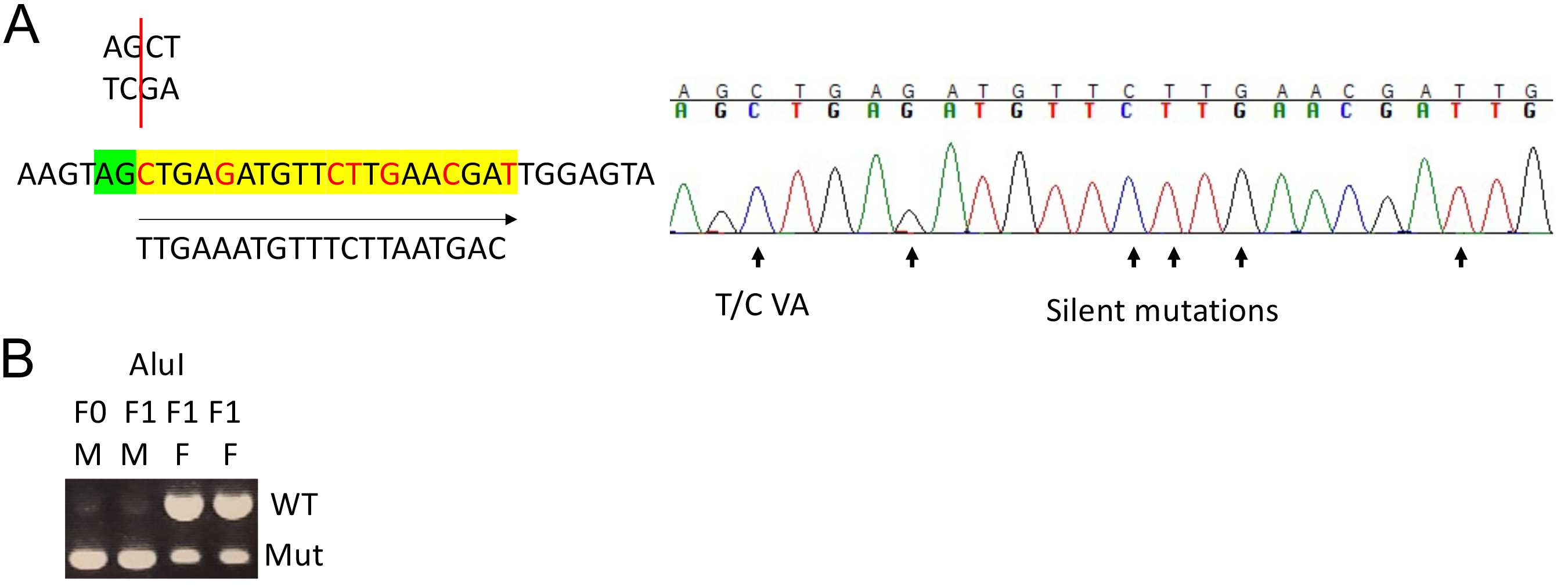

Supplement: Supplementary Figure 4 — Generation and sequence validation of MED14 V769A knockin mice. (A) The sgRNA target sequence is shown with the sgRNA sequence indicated and the region it binds highlighted in yellow, mutations in the protospacer adjacent motif (PAM) to prevent re-cutting are colored red and newly created restriction enzyme screening site is indicated in green. The sequence trace files show PAM mutations, specific V769A mutation, and silent mutations in male founder. An Alu I digest is shown with cleaved bands indicating digestion of the mutant allele. [file Image_4.tif]

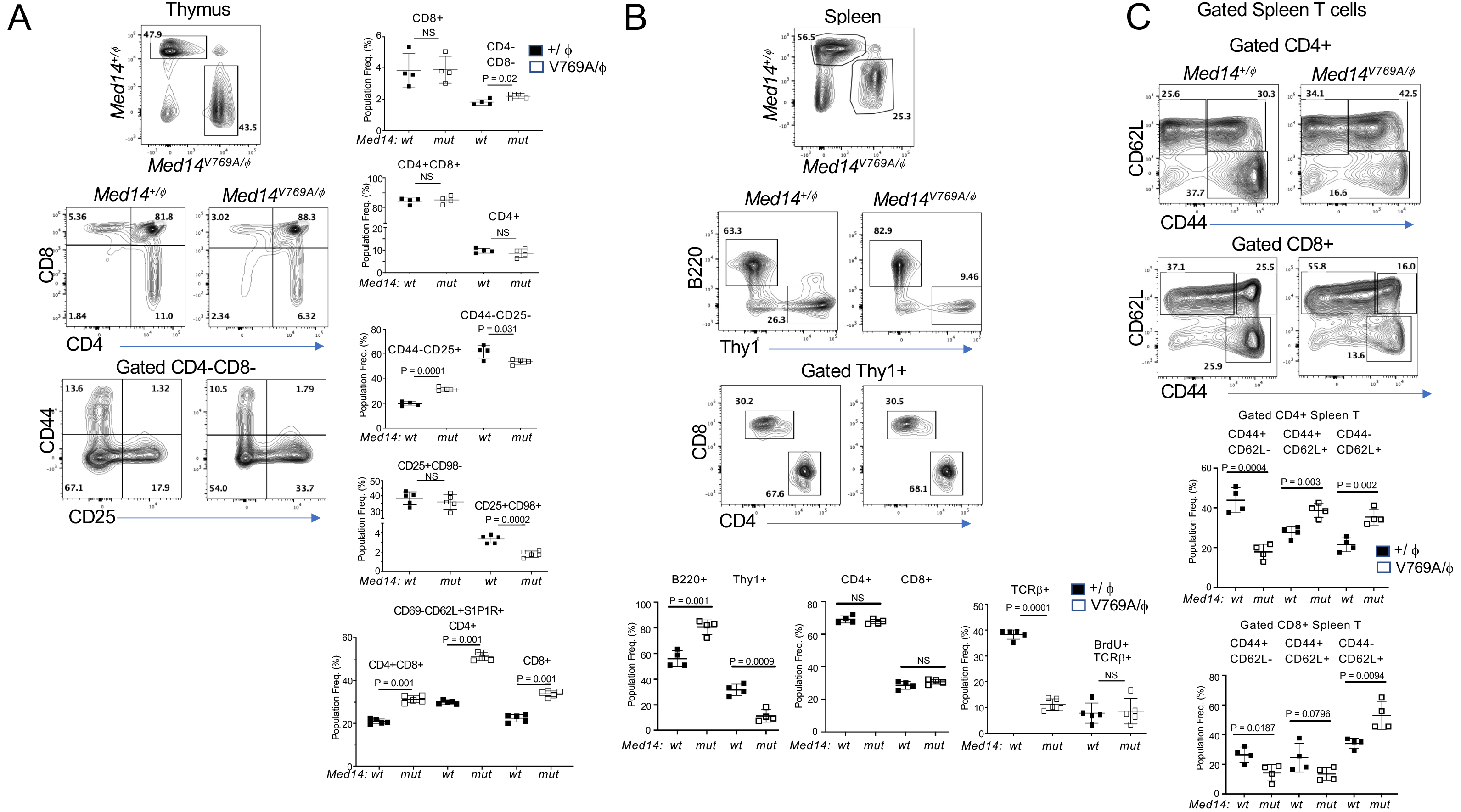

Supplement: Supplementary Figure 5 — Assessment of Med14V769A/ϕ hematopoietic progenitor function by competitive bone marrow transplantation. 100x105 allotype marked wild type (CD45.1) and Med14V769A/ϕ mutant (CD45.2) lineage negative hematopoietic stem and progenitor cells (HSPC) were combined and transferred together into CD45.1 recipients that had been treated with 1100 rads (2x550r, 4h apart) 24h earlier. Recipient mice were placed on antibiotic-treated water (polymyxin B sulfate and neomycin) for 3 weeks and analyzed 6 weeks after transplantation. Single cell suspensions of thymus (A) and spleen (B, C) were stained with CD45.1 and CD45.2 antibodies to distinguish the genotypes of transferred HSPC and with the indicated lineage markers. Bromodeoxyuridine (BrdU) labeling was conducted by staining permeabilized cells after 24h of labeling. Gate frequencies were calculated and depicted as bar graphs of the mean +/- standard deviation. Statistical significance were determined using the t-test. P-values are indicated on the graphs. NS, not significant. [file Image_5.tif]

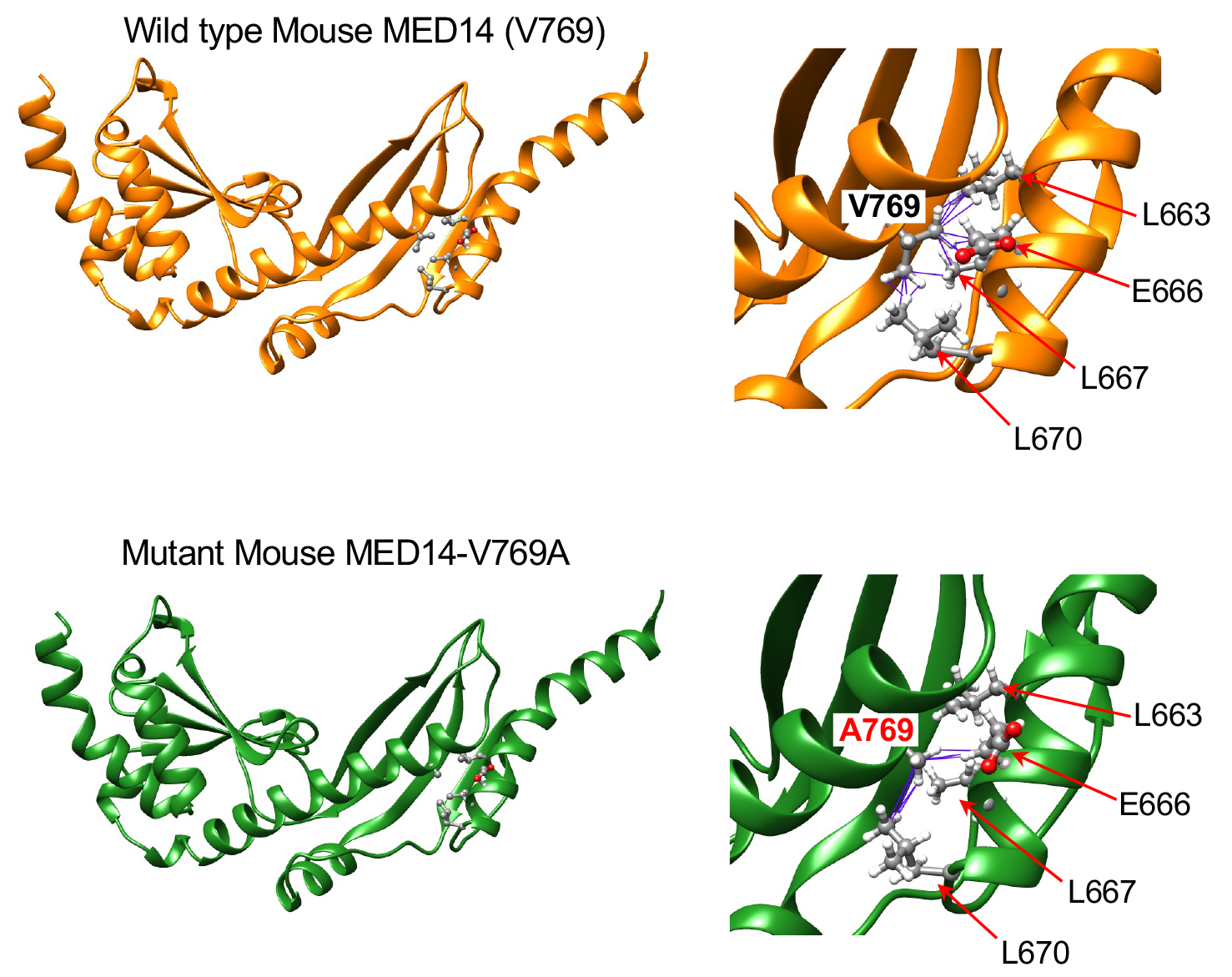

Supplement: Supplementary Figure 6 — Molecular modeling of the wild type and variant mouse MED14 proteins. Two views of wild type (orange) and V769A mutant (green) mouse MED14 are depicted. The right half of each panel shows a zoomed in view of aa 769 with nearby residues on the opposing helix that are capable of making contacts with the A or V769. The top panel shows wild type mouse MED14 V769 from known PDB structure 6W1S chain I, residues 643 to 890. The bottom panel shows the mouse V769A MED14 variant. Hydrophobic contacts are shown with purple lines. [file Image_6.tif]

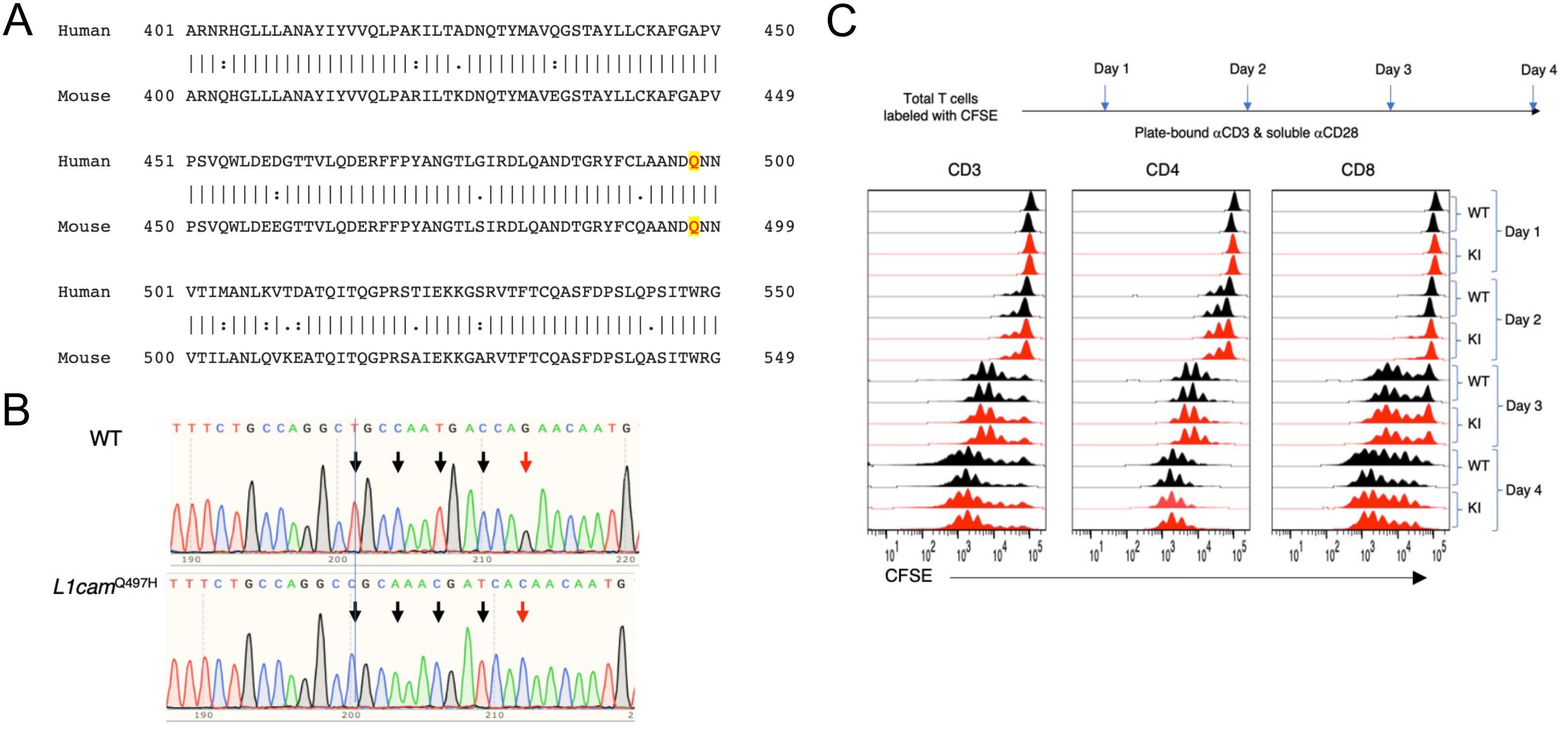

Supplement: Supplementary Figure 7 — Construction and analysis of the the L1CAMQ497H knockin founder mice. (A) Part of human and mouse L1CAM amino acid sequences were aligned and Q498 in human and Q497 in mouse are highlighted and yellow. (B) The founder mouse was identified by PCR using tail gDNA and Sanger sequencing using PCR-Rev primer; black arrows indicate silent mutations introduced to prevent from subsequently cutting by Cas9, which do not change the amino acid and the red arrows indicate a G to C change to make Q to H mutation in mouse. (C) In vitro proliferation assays were performed using splenic T cells isolated from wild type littermates (WT) and L1camQ497H (KI) mice, which were labeled with CFSE, stimulated with 2 μg/ml of plate-bound anti-CD3 and 1 μg/ml of anti-CD28 as indicated. Cell proliferation was determined by CFSE dilution. [file Image_7.tif]
